# Molecular assessment of *atpase6* mutations associated with artemisinin resistance among unexposed and exposed *Plasmodium falciparum* clinical isolates to artemisinin-based combination therapy

**DOI:** 10.1186/1475-2875-11-373

**Published:** 2012-11-09

**Authors:** Sedigheh Zakeri, Samaneh Hemati, Sakineh Pirahmadi, Mandana Afsharpad, Ahmad Raeisi, Navid D Djadid

**Affiliations:** 1 Malaria and Vector Research Group (MVRG), Biotechnology Research Center (BRC), Pasteur Institut, P.O. Box 1316943551, Tehran, Iran; 2 Biology Department, Khatam University, Tehran, Iran; 3 National Programme for Malaria Control, Ministry of Health and Medical Education, Tehran, Iran; 4 Tehran University of Medical Sciences, School of Public Health, Tehran, Iran

## Abstract

**Background:**

Artemisinin-based combination therapy (ACT) is the mainstay of global efforts for treatment of *Plasmodium falciparum* malaria, but decline in its efficacy is the most important obstacle towards malaria control and elimination. Therefore, the present molecular analysis provides information on putative mutations associated with artemisinin resistance in *P*. *falciparum* clinical population unexposed and exposed to artesunate 4 years after adoption of ACT as the first-line anti-malarial therapy in Iran.

**Methods:**

In this study, blood samples (n = 226) were collected from uncomplicated *P*. *falciparum*-infected patients from different health centers of Chabahar district in Sistan and Baluchistan province in the south-eastern part of Iran, during 2003 to 2010. All collected isolates were analysed for putative candidate mutations (TTA) L263E (GAA), (GAA) E431K (AAA), (GCA) A623E (GAA) and (AGT) S769N (AAT) of *pfatpase6* gene using nested PCR/RFLP, followed by sequencing. Furthermore, the gene copy number was assessed by real-time quantitative PCR (RT-qPCR) in the presence of SYBR green.

**Results:**

Neither the *pfatpase6* L263E nor the A623E mutation was detected among all examined isolates. The E431K mutation was found in 23% of the analysed samples unexposed to ACT; however, it was detected in 17.8% (34/191) of *P*. *falciparum* isolates exposed to artesunate after 2007. High frequency of this single nucleotide polymorphisms (SNP) (overall 18.6%) among both examined groups (*X*^2^ test, *P*>0.05) indicated that this SNP should be considered as an unrelated mutation to artemisinin resistance. In contrast, S769N mutation was not detected in unexposed isolates; however, it was found in 2.6% (5/191), four years after introduction of ACT in this malaria setting. Also, detected SNPs were not significantly frequent in both unexposed and exposed examined isolates (*X*^2^ test, *P*> 0.05). Investigation in the copy number of *pfatpase6* gene revealed a similar number of copy (n = 1) as in an isolate sensitive to artemisinin.

**Conclusion:**

Taken together, the results suggest, in particular, that *pfatpase6* S769N gene needs more consideration for its possible association with artesunate resistance among *P*. *falciparum* isolates.

## Background

*Plasmodium falciparum* resistance is one of the most important public health issues in many endemic countries in the recent years. In the absence of a fully-protective anti-malarial vaccine, malaria control heavily relies on the use of drugs for treatment or prophylaxis. However, the increasing failure of the predominantly used and safe drugs, especially chloroquine (CQ) and sulphadoxine-pyrimethamine (SP), has been a serious obstacle towards global malaria control. Consequently, to overcome the resistance problem, the World Health Organization (WHO) and health authorities in malaria endemic countries recommend the use of a therapeutic combination. They recommend the use of artemisinin derivatives in combination with other drugs (ACT) in order to provide a better efficacy and avoidance of resistance nationwide [1,2]. As a result, following increased CQ and SP resistance, artemisinin and its derivatives gradually became the mainstay of falciparum malaria therapy. Besides, previous experiences with other anti-malarial drugs showed that resistance appears after long-term use. Recent reports of the reduced artemisinin sensitivity of *P*. *falciparum* parasites in known foci in Cambodia and Thailand, and new suspected foci in Myanmar and Vietnam may suggest a decline in ACT efficacy [3-5]. Therefore, due to the essential role of ACT in malaria control and elimination, it is crucial to have a regular surveillance of resistance to determine whether resistance to artemisinin has developed or not.

The molecular mechanisms involved in underlying resistance to artemisinin derivatives are not clearly understood and identification of molecular markers would provide insight into these mechanisms. Also, up to the present, artemisinin resistance has not been clinically documented, but it has been reported that the susceptibility of *P*. *falciparum* to artemisinin derivatives is declining in several parts of the world [6]. Laboratory studies have shown that genetically stable and transmissible artemisinin-resistant rodent malaria parasite could be selected through prolonged exposure of drug-sensitive lines to low and increasing levels of artemisinin [7].

Molecular markers are recommended as the earliest technique to detect emerging drug resistance; therefore, the identification and monitoring of genes and mutations, which correlate with resistance to artemisinin and its derivatives, are essential for the evaluation and monitoring of ACT. Previous studies on *P*. *falciparum* have provided evidence that few genes have been associated with artemisinin resistance and these genes are sarcoplasmic/endoplasmic reticulum Ca^2+^-ATPase*6* (*pfatpase6*) [8,9], *pfmdr1*[10-13] and *P*. *falciparum* multidrug resistance protein 1 [14-18]. However, PfATP6 protein might be the major target of artemisinin in *P*. *falciparum*, as the drug completely inhibits the activity of this protein [8,9]. Moreover, recent studies performed with both eukaryotic models and field isolates have proposed that single nucleotide polymorphisms (SNPs) in the *pfatpase6* gene are involved in altered *in vitro* sensitivity to artemisinin or its derivatives [9,19,20]. Also, different studies have previously shown that *pfatpase6* contains a number of SNPs [21-23] and four of them (L263E, E431K, A623E, and S769N) have been reported to be associated with a considerable increase in artemether IC50 [19]. It has been also found that the *pfatpase6* S769N mutation is strongly associated with raised artemether IC50 in *P*. *falciparum* isolates from French Guiana [19]. Besides, it has been suggested that the mutation at L263E codon is involved in the inhibition of PfSERCA by artemisinin [9,19] and allelic exchange study showed reduced susceptibility to artemisinin in parasites expressing the L263E allele [21]. The combination of two additional SNPs, E431K and A623E, was identified in one clinical isolate from Senegal that showed a considerable increase in artemether IC50 [19]. Moreover, other polymorphisms have been identified in *pfatpase6* gene: I89T in Thailand [24], H243Y in Central Africa [25] and silent mutation T2694A in São Tomé and Principe [26]. In 2008, Dahlström and co-workers [22], who studied *P*. *falciparum* isolates from East and West Africa, identified 33 SNPs; three of which were found in a frequency higher than 5% at codons E431K, N569K and A630S.

In October 2007, Iran has switched its first-line treatment policy for uncomplicated falciparum cases to the use of ACT. Nowadays, there is no other anti-malarial drug to replace the artemisinin derivatives; thus, an urgent global priority is needed to prevent potentially emergence and spread of artemisinin resistance. The main goal of the present study was to examine the mutation(s) in the *atpase6* gene of *P*. *falciparum* isolates unexposed and exposed to ACT prior and four years after the adoption of ACT as the first-line anti-malarial drug in hypoendemic malaria settings. In fact, this study attempted to determine whether drug pressure imposed by continuous deployment of ACT causes a potential selection of mutation(s) in *pfatpase6* gene or not, in particular, candidate mutations (TTA)L263E(GAA), (GAA)E431K(AAA), (GCA)A623E(GAA) and (AGT)S769N(AAT).

## Methods

### Study area and sample collection

In this study, blood samples were collected from different health centers of Chabahar district in Sistan and Baluchistan province in the south-eastern part of Iran. In this area, malaria transmission is year-round with two peaks: the first peak occurs during May to August and the second peak mostly happens around October to November and the majority of the cases are infected with *Plasmodium vivax*. Blood samples (n = 226) were collected from uncomplicated *P*. *falciparum*-infected patients, aged from 2 to 70 years old, during 2003 to 2010. The inclusion criteria were the presence of fever for the preceding 48 h (axillary temperature ≥ 37.5°C), mono-infection with *P*. *falciparum*, no intake of anti-malarial drugs for the preceding four weeks and no signs of complications. One milliliter venous blood was collected from *P*. *falciparum*-infected patients prior to treatment based on the National Guideline for treating malaria (CQ from 2003 to 2005, CQ-SP from 2005 to 2007 and SP-artesunate from 2007 onward). All blood samples were collected in a tube containing EDTA, stored at 4°C, and then transported to the main laboratory in Tehran. Furthermore, a written informed consent was obtained from all patients (adults or parents/legal guardians of children) who were participated in the study and an ethics approval was obtained from the Ethical Review Committee of Research of Institut Pasteur, Iran.

### Isolation of parasite genomic DNA from laboratory clones and field isolates of *P*. *falciparum*

Parasite DNA was extracted from 300 μL blood samples of either continuous culture of K1 laboratory strains or field isolates of *P*. *falciparum* using the commercially available DNA purification kit (Promega, Madison, WI, USA), and kept at −20°C until use. DNA of *P*. *falciparum* was detected by nested-PCR amplification of the small sub-unit ribosomal ribonucleic acid (18ssrRNA) genes as described previously [27]. The DNA was resuspended in a Tris-EDTA buffer (10 mM Tris–HCl, pH 8.0, 0.1 mM EDTA) and kept at −20°C until use.

### Nested PCR-RFLP and sequencing

The fragment of *pfatpase6* gene was amplified by nested PCR with oligonucleotide primers and the cycling condition has been demonstrated in Table 1. Briefly, amplification was carried out in a final volume of 25 μL, including 1 μL nest1 PCR product as template, 250 nM primers, 10 mM Tris–HCl (pH 8.3), 50 mM KCl, 2 mM MgCl_2_, 125 μM each of the four deoxynucleotide triphosphates and 0.4 U Taq polymerase (Invitrogen, Carlsbad, CA, USA). Secondary PCR products were digested by four enzymes: ApoI (Fermentas, Vilnius, Lithuania), MBoII (Fermentas, Vilnius, Lithuania), Cac81 (New England Biolab, Beverly, MA, USA) and DdeI (Fermentas, Vilnius, Lithuania) and were used to identify mutations at positions L263E, E431K, A623E, and S769N, respectively (Table 1). Secondary PCR products and digestion fragments were resolved by electrophoresis on a 1.5 and 1.5-3% agarose gel, respectively.

**Table 1:** **PCR/RFLP profiles used for genotyping of the *pfatpase6 *gene**

**PCR Reaction**				**RFLP Reaction**			
Reaction	Primer Sequence (5′→3′)	Ann (Time)	Size	Position	Restriction Enzyme	Product Size	
			(bp)			(bp)	
Nest-1A	F: TTGGTAATAAAACTCCCGC	58 (2’)	948	-	-	-	-
	R: TATTCCTCTTAGCACCACTCC						
Nest-2A	F: TCATCTACCGCTATTGTATG	60 (1’)	775	L263E	Apo I	L: 775	E: 633+142
	R: TCCTCTTAGCACCACTCC			E431K	MBoII	E: 299+241+117+69+49	K: 416+241+69+49
Nest-1B	F: AAGAAGGATAAATCACCAAG	55 (1’)	725	-	-	-	-
	R: AAATACACGTATACCAGCC						
Nest-2Ba	F: TAACCATTCTAATTATACTACAGC**g**CAGG	60 (1’)	141	A623E	Cac8 I	A: 114+27	E: 141
	R: TGTGTTGATGTGGTATTTATTTTATTACCC						
Nest-2Bb	F: AGAACAtTTAGCTTTGCTTATAAAAAA**c**TAA	60 (1’)	164	S769N	DdeI	S: 136+28	N: 164
	R: ATATGGCATAATCTAATTGCTCTTCCTAC						

Based on PCR-RFLP results, 40 samples (n = 20, 2003–2006; n = 20, 2007–2010) were selected for sequencing analysis of the entire *pfatpase6* gene. Therefore, 4032 bp (nt = −66 to 3966 in GenBank, accession no. AB121053) of *pfatpase6* gene, including all reported artemisinin-resistant SNPs, was amplified by using the oligonucleotide primers and the cycling condition has been described in Table 2. Each pair of primers was designed to overlap 70 to 100 bp to cover all sequencing region. The amplified fragments were gel purified using the QIAquick Gel Extraction kit (Qiagen, Hilden, Germany) following the manufacturer’s instructions. Direct sequencing of the DNA fragments was performed in both directions for each PCR product using the dideoxy chain termination procedure (Chemistry V3.1, Applied Biosystems) and also the 3730XL DNA analyzer (Applied Biosystems) by MilleGen sequencing service (Labege, France). Nucleotide and amino acid sequences were aligned and compared with wild-type sequences (accession no. AB121053) using CLUSTAL W.

**Table 2:** **Sequences of oligonucleotide primers and cycling conditions used for full-length *pfatpase6 *sequencing**

**Primer Sequence (5′→3′)**	**Ann (min)**	**Size (bp)**
F1: ATTATATCTTTGTCATTCGTG	55 (1’)	840
R1: TTGTAAAGGTGTTTGAGTATC		
F2: TCATCTACCGCTATTGTATG	60 (1’)	775
R2: TCCTCTTAGCACCACTCC		
F3: AAGTGTTGAGACGTTAGGATG	55 (1’)	698
R3: TTGATGATTGTACAGGTGTTG		
F4: TGGAGACAGTACCGAATTAGC	60 (1’)	814
R4: TCTTCCTACATATTTACGTGGTG		
F5: ATTGTAAAGGTGCACCTGAG	60 (1’)	922
R5: TTACCTAGTGCTGTTGCTGG		
F6: TAGTAATATAGGAGAAGTTGC	60 (1’)	578
R6: TGTATGTTTGTGTGTGTGC		
F7: ATCCACCAGAACATGACG	60 (1’)	760
R7: TCTTGGTTCTTTGCTCTTC		

### Estimation of the copy numbers of *pfatpase6* gene by real-time PCR assay

The gene copy number was assessed by real-time quantitative PCR (RT-qPCR) using a StepOne^Tm^ (Applied Biosystems^TM^) in the presence of SYBR green. Oligonucleotide primers (F: TTGCTGCTATACCAGAAGGATTGC and R:TGTTGTCATTTGATTTGTTGTAAGGG) were manually designed (accession no. AB121053) and verified by both Primer Express® Software for real-time PCR (Version 3.0, Applied Biosystems^TM^) and Gene Runner (Version 3.05). Amplification was carried out in triplicate using the default thermocycler program in a total volume of 20 μl in a 48-well plate (Applied Biosystems^TM^), containing 10 μl 1× SYBR®Green PCR Master Mix (Applied Biosystems, Warrington, UK), 0.75 μl of each of the sense and anti-sense primers (10 μM), 5 μL genomic DNA (20 ng) and 3.5 μL water, with amplicon size of 179 bp. The program was: pre-incubation at 95°C for 10 mins, followed by 40 cycles at 95°C for 15 seconds and at 60°C for 1 min. A PCR negative control with no template was used to check the presence of the dimmers and finally an electrophoresis was used once at the end of the first optimization plate to confirm that a single product of the target gene is present. In this study, the 2^−ΔΔCt^method for relative quantification was used to estimate the copy numbers of *pfatpase6* gene. In this method, to estimate gene copy numbers in unknown samples, at least one calibrator consisting of template DNA with known copies of *pfatpase6* (*P*. *falciparum* K1 with one copy of *atpase6*) and a house-keeping gene of constant copy number in all samples (*pf*-*β*-*tubulin* gene) are required. The formula for the estimation of gene copy numbers in clinical isolates of *P*. *falciparum* is as followed: ^ΔΔ^C_t_ = (C_t_ target gene - C_t_*pf*-*β*-*tubulin*)_unknown sample_ - (C_t_ target gene - C_t_*pf*- *β*-*tubulin*) _K1_. Then, the result for each sample was expressed in N-fold changes in unknown samples (2^−ΔΔCt^). A minimum of two experiments was carried out for each sample and the results were expressed as the N-fold copy number of a given gene relative to *P*. *falciparum* K1, by calculating the mean between the two experiments. In the case where N-fold was between 0.5 and 1.6 (0.5 < N-fold < 1.6), it was accepted that the test sample harbored a single copy of the target gene.

In the present study, β-tubulin as a house-keeping gene was amplified using the primers described previously [28]; F: TCGTCAACTTCCTTTGTGGA and R: TCCCATTCCCACGTTTACAT). To normalize quantitative data in each reaction, all samples were analyzed in triplicate within each LightCycler run and the average value was accepted if the standard deviation was lower than 0.24. The genomic DNA samples used were the same as those used for gene amplification and sequencing. The purity and quantity of DNA were determined by UV spectrophotometry. To determine RT-qPCR efficiencies, standards consisting of 2-fold serial dilutions of genomic DNA were freshly prepared from concentrated stocks for each experiment. Genomic DNAs in the range of 64 to 1 ng were used as quantification standards for the LightCycler calibration curve. Then, amplifications were performed on the same diluted samples using primers for the reference (*pfβtubulin*) and the target genes. The average C_t_ was calculated for both reference and target genes, the ΔC_t_ (C_t_ target gene - C_t_*pfβtubulin*) was determined and the log DNA dilution versus ΔC_t_ was plotted. The efficiency of the PCR should be 90-100% (-3.6 > slope > -3.1). If the absolute value of the slope was close to zero (0.1), the efficiencies of the target and reference genes were similar, and thus the ΔΔC_t_ calculation could be applied.

### Statistics

All statistical analyses were performed using SPSS statistical package (version 16.0). Frequencies of mutations and haplotypes among the groups were compared using the *X*^*2*^ test. *P*<0.05 was considered statistically significant.

## Results

### Sample characteristics

In this investigation, *pfatpase6* gene was successfully amplified in all field samples (n = 226). Of 226 *P*. *falciparum* isolates, 35 samples were collected before the adoption of ACT as the first-line anti-malarial drug in Iran during 2003–2006 with no artemisinin pressure. Also, 49 of 226 samples were collected in 2007, when the artemisinin pressure was very low. Although SP-artesunate was selected to replace CQ-SP in the study areas in year 2007 all malaria settings in Iran undoubtedly used SP-artesunate as the first-line therapy in 2008. Other 142 of 226 samples were collected after the full adoption of SP-artesunate in Iran.

### Prevalence of *pfatpase6* SNPs and allelic distributions

All 226 *P*. *falciparum* samples were successfully genotyped. Surveillance of *pfatpase6* SNPs within positions L263E, E431K, A623E, and S769N indicated no (0/226), 18.6% (42/226), no (0/226), and 2.2% (5/226) prevalence of *pfatpase6*, respectively (Table 3). The majority of the patients (80%, 181/226) was found to carry wild type while single mutant haplotype (L_263_**K**_431_A_623_S_769_), as the second predominant allele, was detected in 16.4% (37/226) studied isolates (Table 3).

**Table 3:** **Frequency distribution of putative SNPs and haplotypes of *pfatpase6 *gene in 226 *P. falciparum *isolates unexposed (<2007) and exposed (>2007) to ACT**

**Gene**	***pfatpase6***				**Year**			**Total**
**Haplotype**	**L263E**	**E431K**	**A623E**	**S769N**	<2007*	2007**	2008-2010***	**n=226 (%)**
					**n=35 (%)**	**n=49 (%)**	**n=142 (%)**	
LEAS	L	E	A	S	27 (77)	44 (89.8)	110 (77.5)	181 (80)
L**K**AS	L	K	A	S	8 (23)	4 (8.2)	25 (17.6)	37 (16.4)
LEA(**SN**)	L	E	A	SN	-	1 (2)	2 (1.4)	3 (1.3)
L(**EK**)AS	L	EK	A	S	-	-	3 (2.1)	3 (1.3)
L**K**A(**SN**)	L	K	A	SN	-	-	1 (0.7)	1 (0.5)
L(**EK**)A(**SN**)	L	EK	A	SN	-	-	1 (0.7)	1 (0.5)

* Unexposed *P. falciparum* isolates to ACT.

** Exposed to ACT with high pressure.

*** Exposed to ACT introduction of ACT as first-line anti-malarial therapy for 4 years.

According to the year of sample collection, before 2007, the prevalent haplotypes were wild type (77%) and L_263_**K**_431_A_623_S_769_ (23%); however, the most frequent haplotype in 2007-2010 was wild type (80.6%) (Table 3). In addition, the number of haplotypes increased from 2 to 6 after ACT adoption in Iran (Table 3). Before and after the introduction of ACT, the prevalence of *pfatpase6* E431K mutation in Sistan and Baluchistan province was 23% (8/35) and 17.8%, (34/191) respectively (Table 3). However, S769N mutation was not detected in any unexposed isolates, but found in 2.6% (5/191) of the exposed isolates (Table 3).

### Sequencing the full length of *pfatpase6* gene

The *pfatpase6* gene of 4032 bp in length was successfully sequenced from the PCR products of 4060 bp by using 7 overlapping paired primers. Both non-synonymous and synonymous mutations were detected at different frequencies in comparison with the reference sequence (accession no. AB121053) as shown in Table 4. Sequencing analysis of samples confirmed the RFLP results. In addition, nine different non-synonyumous mutations at codons 89 (87.5%), 230 (%2.5), 355 (15%), 431 (57.5%), 569 (87.5%), 630 (100%), 683 (97.5%), 769 (6.5%) and 870 (2.5%) were detected in high frequency among both unexposed and exposed *P*. *falciparum* isolates (*X*^2^ test, *P*>0.05) (Table 4).

**Table 4:** **Sequence analysis of SNPs in full-length *pfatpase6 *among 40 *P. falciparum *clinical isolates unexposed (n = 20) and exposed (n = 20) to ACT**

**A: Nonsynonymous**					
**Position**				**≤2007**	**>2007**
**nt**	**aa**		**Codon**		
				**n=20**	**n=20**
266	89	T	AcA	0.25	0
		I	AtA	0.75	1
689	230	I	AtC	0.95	1
		T	AcC	0.05	0
1063	355	I	aTA	1	0.7
		L	tTA	0	0.3
1291	431	E	gAA	0.45	0.40
		K	aAA	0.55	0.60
1707	569	K	AAa	0.25	0
		N	AAt	0.75	1
1888	630	T	aCT	0	0
		H	gCT	0.95	1
		S	tCT	0.05	0
2049	683	K	AAa	0	0.05
		N	AAt	1	0.95
2306	769	S	AgT	1	0.87
		N	AaT	0	0.13
2610	870	F	TTt	1	0.95
		L	TTa	0	0.05
B: **Syonymous**					
981	327	A	GCa	0.05	0
		A	GCt	0.95	1
1449	483	N	AAc	0.2	0
		N	AAt	0.8	1
2694	898	I	Ata	0.8	0.95
		I	ATt	0.2	0.05
2703	901	V	GTa	0.95	1
		V	GTt	0.05	0
3090	1030	K	AAa	0.05	0.05
		K	AAg	0.95	0.95
3093	1031	C	TGc	0.95	1
		C	TGt	0.05	0
3309	1103	Y	Tat	0.95	1
		Y	TAc	0.05	0

nt: nucleotide.

aa: amino acid.

### Copy number of *pfatpase6* gene

In this study, to obtain optimal efficiency, RT-qPCRs were first optimized for each gene by both PCR reagent concentrations and primers and for each gene (including target, house-keeping and reference genes), PCR efficiencies were obtained for about 95% of all cases (data not shown). Regarding the further validity of the RT-qPCR assay, the copy number of *pfatpase6* gene was compared with the laboratory clone *P*. *falciparum* K1 with only one copy number for the mentioned gene. After finding the optimal conditions for *pfatpase6* gene, the estimated gene copy number of all examined field isolates was 1.0 equal to the reference isolate K1.

## Discussion

ACT is the mainstay of global efforts to control *P*. *falciparum* malaria, but suspected failure of artemisinin is the most important obstacle towards malaria control and elimination [5]. At the present, no alternative classes of anti-malarial drugs are available to replace the artemisinin derivatives; hence, an urgent global priority is needed to prevent potentially the emergence and spread of artemisinin resistance. To achieve this goal, determining the molecular mechanism of artemisinin and its derivative resistance is very important. Therefore, the present molecular analysis provides, for the first time, baseline information on putative mutations (L263E, E431K, A623E and S769N) associated with artemisinin resistance in *P*. *falciparum* population unexposed and exposed to ACT in low endemic areas of Iran. In this study, by using nested PCR-RFLP followed by sequencing analysis, neither *pfatpase6* L263E nor A623E mutations was detected among *P*. *falciparum* isolates unexposed (before 2007) and exposed (after 2007) to ACT. In addition, L263E mutation was never found in field isolates [3,19,20,22,23,26,29-33], similar to the present finding, and its association with artemisinin resistance needs more studies to be confirmed.

Concerning E431K, 23% of the isolates harbored this SNP in the collected samples before 2007; however, 17.8% were detected in *P*. *falciparum* samples exposed to artesunate after 2007. The *pfatpase6* E431K mutation has been suggested to be associated with increased artesunate IC50 in Senegal [19] and this SNP has also been reported from African, Asian and South American *P*. *falciparum* isolates. In the present findings we found a high frequency of this SNP among *P*. *falciparum* population unexposed and exposed to artesunate, indicating that this SNP is unlikely associated with artemisinin resistance. Furthermore, the most prevalent haplotype was wild type with a frequency of 77% before 2007 and 80.6% after 2007. The second most frequent haplotype was L_263_**K**_431_A_623_S_769_ with 23% and 15.2% frequency before and after ACT adoption in Iran, respectively. In a previous *in vitro* study, this haplotype with single mutation was sensitive to dihydroartemisinin [33] and the results suggested that the high frequency of E431K among Cameroonian *P*. *falciparum* isolates might be a warning signal for artemisinin drug resistance. Also, although this mutation in high frequency (overall 18.6%) was found among Iranian *P*. *falciparum* isolates, the non-significant prevalence (*X*^2^ test, *P*>0.05) among parasite population unexposed and exposed to ACT suggested that this mutation might have not been selected under artemisinin pressure.

*Pfatpase6* S769N mutation has been reported to affect the PfSERCA activity with increased artemether IC50 and it was originally found in *P*. *falciparum* field isolates from French Guiana [19]. However, it was not reported from China [34], Tanzania [32], Niger [31] and Brazil [35] with a different level of malaria endemicity. In parallel, this mutation was not detected in 35 Iranian *P*. *falciparum* isolates collected during 2001-2002 when CQ was used as first-line anti-malarial therapy [36]. In addition, in the present investigation, further analysis on *P*. *falciparum* isolates collected during 2003-2006 (when CQ-SP was used as the first-line treatment) revealed no S769N mutation. Since ACT was used as the first-line treatment for uncomplicated falciparum malaria in Iran (in 2007), the frequency of this mutation has started to increase (2.6%). Detection of this mutation in only parasite isolates exposed to artesunate might suggest the likely selection of this mutation by artemisinin pressure as the previous study suggested the *pfatpase*6 S769N mutation as a potential molecular marker for *P*. *falciparum* resistance to artemether [19]. Also, the most frequent haplotype before and after ACT adoption was wild type, but the number of haplotypes increased from 2 to 6 in parasite isolates unexposed and exposed to ACT, respectively (Notably, parasites carrying *pfatpase6* S769N mutation). Nonetheless, recent report by Cui *et al*. [37], using allele exchange strategy revealed that 3D7 parasite carrying 769N mutation was still sensitive to artemisinin and its derivatives. This evaluation was done by *in vitro* response of transgenic lines to aforementioned drugs [37]. Although this result argue against the predicted role of *pfatpase* 769N in resistance to artemisinin and its derivatives, but many variables including genetic backgrounds of the parasites may significantly influence on parasite resistance to various anti-malarial drugs [38-42]. Therefore, due to the conflicting data reported by different studies [19,37], present study] the role of this candidate mutation in artemisinin and its derivative resistance needs further study.

In conclusion, the *pfatpase6* genotype and its copy number might make early warning signals for the emergence of ACT resistance and provide baseline data for anti-malarial drug policy. Until now, there is no evidence for assuming that artemisinin resistance has occurred in Iran (Iranian Center for Disease Management and Control, surveillance report, unpublished). Therefore, the present results suggest that, to confirm and distinguish a mutation associated with artemisinin resistance, more studies are required among *P*. *falciparum* population unexposed and exposed to ACT from global malaria endemic setting. However, the *pfatpase6* S769N mutation needs more awareness for its association with artesunate resistance and its value as a molecular marker for monitoring artemisinin resistance among *P*. *falciparum* population remains to be validated in areas where ACT has been used for a longer period. Introducing such a molecular tool could support the national and global malaria control and elimination programmes.

## Competing interests

The authors declare that they have no competing interests.

## Authors’ contributions

SZ designed the study, developed the experimental protocols, supervised field and lab works, finalized the interpretation of the data and wrote down the manuscript. SH, SP and MA performed the lab work and also helped in analysing the data. ND and AR have also contributed to the analysis of the data and critically reading the content of the manuscript. All authors read and approved the final manuscript.
